# Experience of primary care for people with HIV: a mixed-method analysis

**DOI:** 10.3399/bjgpopen19X101665

**Published:** 2019-12-11

**Authors:** Tanvi Rai, Jane Bruton, Meaghan Kall, Richard Ma, Erica Pufall, Sophie Day, Valerie Delpech, Helen Ward

**Affiliations:** 1 Research Associate, School of Public Health, Imperial College London, London, UK; 2 Clinical Research Manager, School of Public Health, Imperial College London, London, UK; 3 Principal Scientist, HIV/STI Department, National Infection Service, Public Health England, London, UK; 4 General Practitioner and NIHR Doctoral Research Fellow, School of Public Health, Imperial College London, London, UK; 5 Research Associate, School of Public Health, Imperial College London, London, UK; 6 Visiting Professor of Anthropology, School of Public Health, Imperial College London, London, UK; 7 Head of HIV Surveillance, Public Health England, London, UK; 8 Professor of Public Health, School of Public Health, Imperial College London, London, UK

**Keywords:** HIV, HIV infections, chronic disease, general practice, empathy, patient satisfaction, social stigma, surveys and questionnaires, qualitative research

## Abstract

**Background:**

Advances in treatment have transformed HIV into a long-term condition (LTC), presenting fresh challenges for health services, HIV specialists, and GPs.

**Aim:**

To explore the experience of people living with HIV (PLHIV) regarding consulting their GPs.

**Design & setting:**

A mixed-method analysis using data from two sources: a nationally-representative survey of PLHIV and a qualitative study with London-based PLHIV.

**Method:**

Univariate logistic regression was used for quantitative data and framework analysis for qualitative data.

**Results:**

The survey had 4422 participants; the qualitative study included 52 participants. In both studies, registration with a GP and HIV status disclosure were high. Similar to general population trends, recent GP use was associated with poor self-rated health status, comorbidities, older age, and lower socioeconomic status. Two-thirds reported a good experience with GPs; a lower proportion felt comfortable asking HIV-related questions. Actual or perceived HIV stigma were consistently associated with poor satisfaction. In the interviews, participants with additional LTCs valued sensitive and consistent support from GPs. Some anticipated, and sometimes experienced, problems relating to HIV status, as well as GPs’ limited experience and time to manage their complex needs. Sometimes they took their own initiative to facilitate coordination and communication. For PLHIV, a ‘good’ GP offered continuity and took time to know and accept them without judgment.

**Conclusion:**

The authors suggest clarification of roles and provision of relevant support to build the confidence of PLHIV in GPs and primary care staff to care for them. As the PLHIV population ages, there is a strong need to develop trusting patient–GP relationships and HIV-friendly GP practices.

## How this fits in

Existing literature reports that PLHIV are often dissatisfied with primary care services. This study found that PLHIV value good access, continuity, and communication between primary and secondary care, and it is important to them to feel confident that GPs will have some understanding of their condition. GPs can improve the lives and experiences of PLHIV by using similar models to those used for other LTCs, and by employing existing skills of empathy and support. Structural improvements (assurance of confidentiality, better continuity and communication) and training need not be resource-intensive and can deliver quality care for PLHIV.

## Introduction

A strategy of earlier diagnosis and prompt treatment with modern antiretroviral therapy (ART) that achieves viral suppression rapidly has transformed the lives of PLHIV, enabling them to have a normal life expectancy.^1^ In 2018, 40% of PLHIV accessing specialist care in England were aged >50 years;^2^ Public Health England estimates this to rise to >50% by 2028.^3^ Health services now face the challenge of meeting the needs of an ageing cohort of PLHIV as they develop age-related comorbidities.

HIV clinics traditionally managed all healthcare needs of PLHIV, but modern ART has minimised HIV-related complications and those with stable disease are likely to develop age-related LTCs such as cardiovascular diseases, malignancies, and frailty, which may or may not be HIV-related.^4^ Management of these LTCs falls outside the scope of HIV clinics, while GPs and primary care teams have experience and competence in managing LTCs and could have a greater role in the care of PLHIV. Indeed, the British HIV Association recommends PLHIV register with a GP to access primary care and prevention measures.^5^

A survey of over 1300 PLHIV explored their experiences of London HIV services and reported that most were registered with, and had disclosed their HIV status to, GPs.^6^ The qualitative sub-study identified barriers to primary care use including: concerns regarding GP’s HIV knowledge; poor communication between primary and specialist care; difficulties negotiating continuity of care, and concerns about confidentiality, discrimination, and discussing sexual matters.^7,8^ GPs themselves have concerns about their HIV expertise; recognising when to refer to specialist services; knowledge of possible drug interactions; time pressures; and low HIV caseloads making it difficult to maintain skills.^9^ GPs and HIV specialists involved in ‘shared care‘ concluded that close collaboration between HIV-interested GPs and specialists was key, but only feasible in practices with high HIV caseloads.^10^ There are no data that report PLHIV’s experience of GP services nationally, and most qualitative studies recommend little beyond GP education and system-level changes. This research aims to fill this gap by: a) reporting prevalence of GP registration, disclosure of HIV status, and satisfaction with GP services by quantitative analysis of a nationally-representative sample of PLHIV; and b) examining personal experiences of PLHIV consulting their GPs, to identify practical steps for improvement from a qualitative research study with PLHIV in London. Previous research from the latter, qualitative study has described PLHIV perspectives of going through the treatment cascade,^11^ and how they view the contemporary metanarrative of HIV as a chronic condition.^12^ This article focuses specifically on PLHIV’s views and experiences of using primary care services.

## Method

### Quantitative study: Positive Voices survey

Details of study participants, recruitment methods, and the wider content of the survey are presented elsewhere.^13^ Briefly, a representative sample of people attending 73 HIV clinics in England and Wales were invited to take part. The survey was self-completed, online or on paper, and participants received a £5 high street voucher. The sample was weighted using population data (age, sex, ethnicity, geography, risk group) from national surveillance records, representative of all PLHIV accessing care in England and Wales. Survey responses were linked to treatment and viral load data in the national HIV surveillance system (HIV and AIDS Reporting System). The survey included a section about use of primary care services. It asked participants about GP registration; disclosure of HIV status; recent GP contact (past 3 months); and four 5-item Likert scale patient-reported experience measures (PREMs) on experience of HIV care from their GP; namely, their GP’s HIV knowledge, involvement in HIV care, communication with specialist, and their own comfort in asking their GP questions about HIV. All statistical analyses were conducted in Stata/MP (version 15.1). The research team carried out descriptive univariate analysis looking at the association between participant characteristics and GP registration, disclosure, recent contact, and the four PREMs, using a standard alpha of <0.05.

### Qualitative study: the journeys of PLHIV

As part of a larger study exploring the experiences of PLHIV across the 40-plus year history of HIV and antiretroviral treatment,^11,12^ participants were recruited from two large HIV clinics in London; details of the methods are published elsewhere.^11^ Briefly, participants were recruited between September 2014–April 2015 using a sampling frame that identified four ‘HIV generations‘: generation 1 (diagnosed pre-1996, before ART), generation 2 (1997–2005, complex ART), generation 3 (2006–2012, simpler ART), and generation 4 (recently diagnosed, 2013 onwards). Within each generation, people were recruited across a range of characteristics (age, gender, sexual orientation, and ethnicity).

Four researchers, three female and one male (see Acknowledgements) conducted one-to-one, semi-structured interviews at a time and place convenient to the participant. Interviews lasted 40–90 minutes and were recorded digitally. The topic guide was developed with the broader aim of exploring ongoing experiences of living with HIV and has been described in previous publications from the study.^11,12^ Within these interviews, participants were asked specifically about interactions with GPs. GPs were also mentioned in other contexts; for example, when discussing HIV diagnosis or ongoing health management.

Data were analysed using framework methods,^14^ using both a case- and theme-based approach, facilitated by Nvivo (version 10) software. All interviews were read repeatedly by two members of the research team, and every instance where GPs were mentioned was coded under an overall theme regarding participant–GP interaction; the interviews were further analysed and agreed with a third coder. All sub-themes regarding participant–GP interaction were compared within each interview in terms of wider experiences of living with HIV and across interviews until no new insights emerged.

### Patient and public engagement and involvement

Findings of the Positive Voices survey have been discussed with participants and clinicians at meetings around the country, including with a group of service users from Positively UK. For the qualitative study, a focus group of PLHIV was held to advise on the study design and topic guide, and the findings have been disseminated to patient and public groups in the UK and abroad at international conferences and meetings.

## Results

### Quantitative study

The Positive Voices 2017 survey recruited 4422 participants from 73 HIV clinics in England and Wales between January–September 2017, giving a response rate of 50.8% (*n* = 4422/8700, Table 1). GP registration rate was 97.5%, with no significant variation by any measured demographic variables. Of these, 93.8% had disclosed their HIV status to their GP. GP disclosure was high (>85%) across all groups examined, though certain groups were significantly (*P*<0.05) less likely to disclose: younger adults (88.8% among those aged 15–34 years versus 95.5% aged ≥50); those with higher educational attainment (91.4% with post-graduate degrees versus 98.6% with primary school education or less); those in good health (92.9% in those who self-rated health as good or very good versus 98.8% who self-rated as bad or very bad; 89.8% in those with no other comorbidities versus 98.3% in those with ≥4 comorbidities). One in six (16.8%) reported avoiding health care due to their HIV, and this was also associated with GP non-disclosure (89.8% who avoided versus 94.8% who never avoided).

**Table 1.** Factors associated with GP disclosure and recent attendance among PLHIV registered with a GP

	**Denominator**	**Disclosed to GP**	**Attended GP in** **the last** **3** **months**
	Weighted	Unweighted	%	OR (95% CI)	*P* value	%	OR (95% CI)	*P* value
**Overall**	83 668	4422	93.8			58.0		
								
**Gender and sexuality** ^a^					0.01			0.001
Gay and bisexual men	38 300	2264	92.8	1		55.6	1	
Heterosexual men	19 217	846	93.3	1.08 (0.75 to 1.56)		56.3	1.03 (0.87 to 1.23)	
All women	26 150	1208	95.6	1.68 (1.18 to 2.38)		62.0	1.31 (1.14 to 1.50)	
**Age, years**					<0.001			0.003
15–34	10 535	490	88.8	1		51.5	1	
35–49	36 413	1911	93.5	1.81 (1.28 to 2.57)		56.3	1.21 (0.95 to 1.53)	
≥50	36 720	1915	95.5	2.63 (1.97 to 3.51)		61.3	1.48 (1.15 to 1.91)	
**Residence**					0.064			0.002
London	41 364	2146	92.1	1		58.6	1	
South of England	12 186	693	94.4	1.44 (0.88 to 2.38)		52.3	0.76 (0.61 to 0.94)	
Midlands, East of England, and Wales	16 883	885	95.2	1.70 (1.05 to 2.75)		57.9	0.95 (0.76 to 1.19)	
North of England	13 235	698	95.9	2.02 (1.01 to 4.05)		62.0	1.14 (0.97 to 1.34)	
**Education level**					<0.001			<0.001
Primary or less	5562	267	98.6	1		64.9	1	
Upper secondary	32 403	1603	94.5	0.23 (0.09 to 0.62)		59.1	0.79 (0.61 to 1.03)	
Technical & vocational	8244	410	95.7	0.31 (0.11 to 0.83)		64.8	1.00 (0.69 to 1.45)	
Bachelor’s degree	24 268	1167	92.8	0.18 (0.07 to 0.44)		54.6	0.65 (0.47 to 0.90)	
Postgraduate degree	13 191	662	91.4	0.14 (0.06 to 0.35)		53.5	0.62 (0.47 to 0.82)	
**Employment status**					<0.001			<0.001
Employed	54 240	2736	92.2	1		51.0	1	
Unemployed	9108	421	95.6	1.84 (1.03 to 3.28)		66.5	1.90 (1.52 to 2.38)	
Economically inactive^b^	20 320	1055	97.4	3.16 (1.84 to 5.43)		71.9	2.46 (2.02 to 3.0)	
**Non-HIV comorbidities**					<0.001			<0.001
None	25 073	1272	89.8	1		42.7	1	
One	19 573	997	92.0	1.29 (0.80 to 2.07)		51.6	1.46 (1.20 to 1.76)	
Two	13 547	724	95.2	2.27 (1.45 to 3.56)		61.2	1.98 (1.64 to 2.40)	
Three	9795	530	97.0	3.69 (2.01 to 6.78)		70.9	3.19 (2.59 to 3.92)	
Four or more	15 679	866	98.3	6.39 (2.93 to 13.95)		79.1	4.92 (3.92 to 6.18)	
**Self-rated health**					0.012			<0.001
Good/very good	60 737	3161	92.9	1		52.4	1	
Fair	18 037	943	95.7	1.68 (1.03 to 2.73)		68.6	1.99 (1.65 to 2.39)	
Bad/very bad	4894	255	98.8	6.22 (1.27 to 30.53)		84.9	5.16 (3.47 to 7.69)	
**Money for basic needs**					0.004			<0.001
Yes, always	39 142	2019	92.6	1		50.6	1	
Not always	44 526	2075	94.9	1.48 (1.114 to 1.93)		64.2	1.73 (1.51 to 1.98)	
**ART status**					0.004			0.072
On ART	81 778	4294	94.0	1		58.3	1	
Not on ART	1890	99	86.7	0.42 (0.23 to 0.75)		47.9	0.66 (0.41 to 1.04)	
**Viral suppression** ^**c**^					0.987			0.053
Yes	81 333	3539	93.9	1		57.4	1	
No	2335	98	93.9	0.99 (0.39 to 2.56)		67.7	1.53 (0.99 to 2.35)	
**Year of HIV diagnosis**					<0.001			<0.001
Pre-1996	10 298	545	97.6	1		66.1	1	
1996–2005	31 871	1593	94.8	0.45 (0.26 to 0.77)		59.3	0.75 (0.60 to 0.94)	
2006–2017	41 499	2149	92.1	0.29 (0.16 to 0.53)		54.8	0.62 (0.49 to 0.78)	
**Worried about discrimination in health care due to HIV**					0.833			0.005
Never	54 169	2733	94.1	1		56.8	1	
>1 year ago	16 210	840	94.3	1.01 (0.69 to 1.49)		56.2	0.97 (0.83 to 1.13)	
In the last year	13 289	670	93.5	0.89 (0.59 to 1.36)		64.3	1.36 (1.13 to 1.63)	
**Avoided health** **care due to HIV**					<0.001			0.159
Never	68 465	3442	94.8	1		57.7	1	
>1 year ago	7035	345	90.6	0.52 (0.34 to 0.79)		54.1	0.85 (0.68 to 1.06)	
In the last year	8168	400	89.1	0.44 (0.28 to 0.70)		60.3	1.11 (0.92 to 1.33)	

ART = antiretroviral therapy. CI = confidence intervals. OR = odds ratio.

a Transmen and transwomen are included in the gender groups with which they self-identified. Due to small numbers, those who identify as non-binary/in another way (representing 0.5% of Positive Voices responders) are excluded from the binary gender breakdown. Gay and bisexual men are identified as MSM (men who have sex with men) in the main text.

b Economically inactive includes individuals who are not in employment or unemployed, including those who are retired, looking after family, long-term sick, carers, students, and those whose immigration status does not permit them to do paid work.

c Viral suppression defined as VL <200 copies/ml in last reported viral load result in 2017.

Over half (58.0%) had attended their GP surgery in the previous 3 months, with recent GP attendance most strongly associated with poor self-rated health (odds ratio [OR] 5.16, 95% confidence intervals [CI] = 3.47 to 7.69; *P*<0.001) and increasing burden of diagnosed non-HIV related comorbidities (*P*<0.001; Table 1).

Experiences with GPs across the four PREMs varied widely, but around two-thirds agreed with the PREM statements on GP’s HIV knowledge (GP knows enough about HIV; 64.7%), GP’s HIV involvement (GP is as involved in HIV care as they wanted; 65.1%), and GP–specialist communication (HIV specialist and GP communicate well; 64.1%), as shown in Table 2. A slightly lower proportion said they felt comfortable asking their GP questions about HIV (58.9%). Across the four PREM statements, common factors significantly associated with high levels of agreement were older age, lower educational attainment, being in full- or part-time employment, and good or very good health status (all *P*<0.05). Heterosexual men and women reported higher agreement with statements on GP’s HIV knowledge and GP–specialist communication compared to men who have sex with men (MSM); (*P*<0.001), whereas MSM were more likely to agree their GP is as involved as they want them to be (*P*<0.001).

**Table 2.** Factors associated with patient-reported experience measures^a^ among PLHIV registered with their GP

	**GP knows** **enough about HIV**	**GP is as involved as I want them to be in HIV care**	**Good communication** **between GP and HIV specialist**	**Comfortable** **asking GP questions about HIV**
	**%** **agreement**	**O** **R** **(** **95%** **CI** **)**	** ***P*** **value**	**%** **agreement**	**O** **R** **(95% CI)**	***P*** **value**	**%** **agreement**	**OR** **(95% CI)**	***P*** **value**	**%** **agreement**	**O** **R** **(95% CI)**	** *P* **value**
**Overall**	64.7			65.1			64.1			58.9	
**Gender and sexuality** ^**b**^			<0.001			<0.001			<0.001			0.018
Gay and bisexual men	57.6	1		69.2	1		57.3	1		57.6	1	
Heterosexual men	73.1	1.98 (1.65 to 2.37)		67.4	0.94 (0.78 to 1.13)		69.8	1.72 (1.41 to 2.10)		65.4	1.39 (1.11 to 1.74)	
All women	69.0	1.63 (1.36 to1.96)		58.5	0.63 (0.52 to 0.74)		70.0	1.73 (1.41 to 2.11)		56.2	0.93 (0.82 to 1.08)	
**Age** **, years**			<0.001			<0.001			<0.001			<0.001
15–34	56.3	1		57.6	1		52.3	1		48.3	1	
35–49	62.5	1.27 (1.02 to 1.60)		61.3	1.15 (0.93 to 1.43)		61.5	1.44 (1.12 to 1.86)		55.2	1.31 (1.01 to 1.69)	
≥50	69.3	1.74 (1.36 to 2.22)		72.0	1.86 (1.43 to 2.43)		70.0	2.10 (1.62 to 2.74)		65.9	2.05 (1.57 to 2.67)	
**Residence**			0.279			0.039			<0.001			0.058
London	65.0	1		62.3	1		59.1	1		57.3	1	
South of England	61.9	0.88 (0.74 to 1.05)		68.3	1.29 (1.03 to 1.61)		63.0	1.18 (0.93 to 1.51)		59.7	1.03 (0.79 to 1.33)	
Midlands, East of England, and Wales	64.4	0.99 (0.81 to 1.20)		64.9	1.11 (0.87 to 1.41)		69.0	1.55 (1.26 to 1.90)		58.9	1.01 (0.84 to 1.22)	
North of England	66.9	1.10 (0.90 to 1.20)		69.9	1.38 (1.05 to 1.81)		71.4	1.72 (1.44 to 1.51)		63.6	1.31 (1.06 to 1.61)	
**Education level**			0.033			0.158			<0.001			<0.001
Primary or less	71.2	1		69.3	1		71.1	1		66.7	1	
Upper secondary	66.3	0.80 (0.60 to 1.07)		66.7	0.87 (0.66 to 1.14)		65.9	0.81 (0.55 to 1.20)		62.1	0.84 (0.62 to 1.14)	
Technical & vocational	66.6	0.80 (0.58 to 1.10)		67.6	0.89 (0.60 to 1.32)		68.1	0.89 (0.56 to 1.40)		64.0	0.90 (0.65 to 1.24)	
Bachelor’s degree	63.0	0.69 (0.51 to 0.93)		64.8	0.80 (0.61 to 1.04)		63.3	0.73 (0.50 to 1.06)		55.5	0.64 (0.47 to 0.86)	
Postgraduate degree	58.0	0.55 (0.37 to 0.82)		62.7	0.72 (0.51 to 1.02)		54.7	0.51 (0.34 to 0.76)		51.5	0.54 (0.37 to 0.78)	
**Employment status**			0.011			<0.001			<0.001			<0.001
Employed	62.2	1		63.9	1		60.7	1		56.8	1	
Unemployed	68.2	1.32 (0.97 to 1.78)		60.5	0.86 (0.66 to 1.12)		64.4	1.15 (0.91 to 1.45)		60.4	1.16 (0.95 to 1.41)	
Economically inactive^c^	67.5	1.26 (1.09 to 1.47)		71.2	1.37 (1.13 to 1.65)		70.5	1.53 (1.28 to 1.82)		63.4	1.30 (1.14 to 1.48)	
**Non-HIV comorbidities**			<0.001			0.328			0.358			0.281
None	67.4	1		62.4	1		62.8	1		60.2	1	
One	61.6	0.84 (0.72 to 0.99)		65.0	1.10 (0.87 to 1.40)		62.5	1.07 (0.85 to 1.33)		58.5	0.98 (0.84 to 1.14)	
Two	66.1	0.93 (0.76 to 1.15)		67.3	1.23 (0.93 to 1.62)		64.2	1.10 (0.89 to 1.35)		61.0	0.98 (0.79 to 1.23)	
Three	67.9	1.09 (0.82 to 1.45)		69.4	1.34 (1.00 to 1.80)		67.0	1.23 (0.93 to 1.63)		58.4	0.99 (0.79 to 1.24)	
Four or more	60.0	0.77 (0.63 to 0.94)		66.0	1.17 (0.87 to 1.57)		65.8	1.20 (098 to 1.46)		56.4	0.87 (0.73 to 1.03)	
**Self-rated health**			<0.001			0.007			0.351			0.023
Good/very good	66.9	1		66.2	1		64.7	1		60.7	1	
Fair	60.3	0.75 (0.63 to 0.90)		64.2	0.91 (0.73 to 1.14)		63.5	0.94 (0.80 to 1.11)		55.5	0.81 (0.68 to 0.96)	
Bad/very bad	55.2	0.61 (0.45 to 0.83)		57.8	0.70 (0.56 to 0.87)		60.2	0.83 (0.64 to 1.07)		54.0	0.76 (0.59 to 0.97)	
**Money for basic needs**			0.004			<0.001			0.018			0.271
Yes, always	62.3	1		70.1	1		61.3	1		60.1	1	
Not always	66.5	1.20 (1.06 to 1.36)		61.3	0.66 (0.55 to 0.80)		66.3	1.23 (1.04 to 1.46)		58.0	0.92 (0.80 to 1.07)	
**ART status**			0.737			0.611			0.936			0.259
On ART	64.7	1		65.1	1		64.0	1		58.8	1	
Not on ART	63.2	0.92 (0.55 to 1.52)		68.5	1.16 (0.64 to 2.11)		63.9	0.98 (0.58 to 1.65)		66.1	1.35 (0.80 to 2.29)	
**Viral suppression** ^**d**^			0.517			0.039			0.209			0.566
Yes	63.7	1		65.1	1		63.7	1		58.5	1	
No	66.9	1.17 (0.72 to 1.92)		51.7	0.58 (0.34 to 0.97)		55.9	0.72 (0.43 to 1.20)		54.7	0.86 (0.52 to 1.44)	
**Year of HIV diagnosis**			0.013			0.038			0.053			0.191
Pre-1996	59.2	1		69.0	1		64.1	1		58.3	1	
1996–2005	65.6	1.29 (1.07 to 1.55)		62.7	0.74 (0.59 to 0.94)		66.1	1.09 (0.94 to 1.26)		57.1	0.95 (0.75 to 1.20)	
2006–2017	64.6	1.26 (1.07 to 1.49)		65.7	0.86 (0.69 to 1.08)		61.4	0.90 (0.77 to 1.05)		59.9	1.08 (0.87 to 1.34)	
**Worried about discrimination in health care due to HIV**			<0.001			<0.001			<0.001			<0.001
Never	70.5	1		69.4	1		67.6	1		65.8	1	
>1 year ago	55.7	0.52 (0.42 to 0.64)		61.1	0.68 (0.57 to 0.81)		59.7	0.70 (0.60 to 0.83)		50.4	0.52 (0.44 to 0.62)	
In the last year	49.8	0.42 (0.34 to 0.51)		54.3	0.52 (0.40 to 0.69)		53.9	0.56 (0.45 to 0.70)		41.4	0.37 (0.30 to 0.45)	
**Avoided seeking health** **care when needed due to HIV**			<0.001			<0.001			<0.001			<0.001
Never	67.6	1		68.3	1		65.7	1		62.3	1	
>1 year ago	53.0	0.55 (0.42 to 0.73)		59.3	0.67 (0.51 to 0.90)		56.6	0.67 (0.51 to 0.89)		46.7	0.53 (0.42 to 0.66)	
In the last year	43.6	0.37 (0.31 to 0.45)		46.5	0.40 (0.33 to 0.50)		52.7	0.58 (0.44 to 0.77)		36.3	0.35 (0.28 to 0.43)	

ART = antiretroviral therapy. CI = confidence intervals. OR = odds ratio. PLHIV = people living with HIV.

^a^The four 5-item Likert scale patient-reported experience measures (PREMS) on experience with their GP as relates to their HIV: 1)'My GP knows enough about my HIV condition and treatment'; 2) 'My GP is as involved as I want them to be with my HIV care'; 3) 'My HIV specialist and my GP communicate well regarding my health'; 4)'I am comfortable asking my GP questions about HIV'. Responses of ‘Strongly Agree‘ and ‘Agree‘ were combined, and ‘Disagree‘, ‘Strongly Disagree‘, and ‘Don’t know or Not applicable‘ were combined.

^b^Transmen and transwomen are included in the gender groups with which they self-identified. Due to small numbers, those who identify as non-binary/in another way (representing 0.5% of Positive Voices respondents) are excluded from the binary gender breakdown. Gay and bisexual men are identified as MSM (men who have sex with men) in the main text.

^c^Economically inactive includes individuals who are not in employment or unemployed, including those who are retired, looking after family, long-term sick, carers, students, and those whose immigration status does not permit them to do paid work.

^d^Viral suppression defined as VL <200 copies/ml in last reported viral load result in 2017.

The most negative experiences with GPs were consistently among participants who reported experienced or internalised HIV-related stigma (worried about discrimination in healthcare settings and/or avoided health care because of HIV), particularly if they had experienced this stigma in the past year. People who avoided health care in the past year due to their HIV status were significantly less likely to feel comfortable asking their GP questions about HIV compared to those who had never avoided health care (OR 0.35, 95% CI = 0.28 to 0.43; *P*<0.001). Similarly, they were less likely to agree with PREM statements on GP HIV knowledge (OR 0.37, 95% CI = 0.31 to 0.45; *P*<0.001), GP HIV involvement (OR 0.40 95% CI = 0.33 to 0.50; *P*<0.001), and GP–specialist communication (OR 0.58, 95% CI = 0.44 to 0.77; *P*<0.001) as shown in Table 2.

ART and viral suppression were not significantly associated with GP attendance or the four PREM statements; however, nearly all (95.6%) participants were on ART and had a suppressed viral load. Patient experience with GPs did not vary by region of residence, time since HIV diagnosis, or presence of non-HIV related comorbidity.

### Qualitative study

A total of 52 adults aged 25–70 years were interviewed. There were 37 MSM, 4 heterosexual men, and 11 heterosexual women.^11^ All names provided are pseudonyms.

#### GP registration

Most participants were registered with a GP. Four men were not registered: two were just registering after changed circumstances, and two used non-NHS GPs. Participants did not visit their GP as often as the HIV clinic unless they had chronic, non-HIV LTCs needing regular supervision. Younger, newly diagnosed participants perceived themselves to be in good health and rarely visited their GPs, whereas unwell (either due to HIV or comorbidities) participants were receiving care from specialist services rather than GPs.

Most participants felt obliged to register because this was recommended by their clinics. Those with other LTCs felt it was in their best interest because some non-HIV related services previously provided by HIV centres could now only be accessed via GPs (such as psychological therapies, statins, influenza vaccination, and cervical cytology).

#### HIV disclosure, confidentiality, and stigma

Echoing the quantitative findings, most participants had disclosed their HIV status to their GP. One participant had not, fearing this would shift his care from the HIV clinic to his GP. Two others delayed telling their GP until they started ART: disclosure symbolised final acceptance of being HIV-positive for one (quoted below); the other had used his GP for mental health problems and worried that disclosing his HIV as well could jeopardise future employment.

*‘* *I didn't want my GP to know that I was HIV, not right now. I wanted to tell them, I know it sounds stupid, but when I start the med*s […] *the whole idea of having HIV will kick in. I think it will sink in then. I'll just accept it. I do accept it now, but it's not something that I think about every day.* *’* (Sunny, MSM, 33 years)

Although HIV disclosure to GPs was common, concerns about confidentiality were widespread, particularly with regards to non-clinical staff such as receptionists knowing their status. Participants wondered if it could be disclosed to employers or other people they knew, especially within small communities. The present authors have previously reported that maintaining secrecy about their status was vital to PLHIV coping with their status:^12^

*‘I was a bit scared about telling my GP because I didn’t want, with my job and health insurance and all these kind of things, it* [to] *be on my notes.’* (Anthony, MSM, 35 years)

*‘* *…* *it’s awkward because I know, my daughter swims with the receptionist, and I've always wondered, “Does she know?* *”’* (Sarah, heterosexual female, 53 years)

David recalled his astonishment when he saw his electronic patient record was flagged with his HIV status:

*‘* *… he* [the GP] *just turned the screen and he said,* *“* *There’s a marker on about your health.* *”* *I said,* *“* *What’s that?* *”* *and he said,* *“* *Well, this.* *”* *On the screen it said,* *“* *HIV positive,* *”* *on there and he said he puts it on there because of the health and safety of his staff. I thought,* *“* *Oh God, is it as blatant as that?* *”* *but we are going back about 5 years ago, things might have changed a bit …* ‘ (David, MSM, 59 years)

#### Empathy and support

Many participants described how receiving consistent support from their HIV clinic, medically and psychologically, from the beginning of their ‘HIV journey‘ and at their most vulnerable, had embedded a deep sense of trust in them (see also previous publication from this research).^11^ To replicate a similar level of understanding with their GP, they needed to feel confident that their GP was willing to listen, take their problems seriously, and help. GPs who demonstrated kindness and support stood out in participants’ accounts. For example, Keith remembered feeling reassured when his GP was warm and empathetic on their first meeting:

*‘* *I then found a GP, which was not far from me, which was great. I went in there, with my carer with me* *and I said,* *“* *This is what’s happening.* *”* *He said,* *“* *That’s okay, let me see what I can do to make life easier.* *”* *I’ll never forget him saying that.* *’* (Keith, MSM, 50 years)

Similarly some patients recalled having personal issues, mental health problems, and crises which their GPs helped them through. Those with other LTCs reported positive relationships with their GPs.

However, participants who perceived an excessive emphasis on their HIV-positive status hesitated to access GP services. Susan found it distressing that her GP seemed to attribute every symptom she presented with to be potentially HIV-related:

*‘* *I hate going to my GP … everybody just looks at me as walking HIV.* *’* (Susan, heterosexual female, 53 years)

#### Establishing confidence and continuity of care

Sometimes the HIV clinic recommended ‘HIV-friendly‘ GPs, giving participants the confidence to register. One participant reported that when he registered at the surgery he was assigned a particular doctor:

*‘* *They didn’t really know what to do initially, but then I got a phone call that there’s a specific doctor that deals with HIV cases within the surgery. So, they’ve been good* *.* *’* (Colin, MSM, 35 years)

Participants who saw the same GP each visit developed a trusting relationship, felt confident in their care, and felt their GP demonstrated an interest in and/or knowledge about HIV. Some participants described having easy or ‘open-door‘ access to their GP:

*‘* *I think it’s one of those ones where I’m quite lucky I’ve got a good practice and, actually, I’ve got somebody there that if there is something a bit more personal, I can talk to him, and also I can talk to him officially or unofficially* *.’* (Brian, MSM, 39 years)

However, the perception that GP surgeries were ‘family friendly‘ spaces sometimes was a barrier to discussing medically-relevant but socially-stigmatised activities relating to drinking, drug-taking and sex. Unlike their experience at the HIV clinic, many participants felt revealing details to their GP about past behaviour or future intentions could provoke alarm or judgment:

*‘* *I feel much more comfortable sitting with* [HIV clinic doctor] *discussing and asking questions about oral sex, other types of sex and anything about my sexual health than I would with my GP. He’s not my family GP, but he’s a family GP. I want to go to him when I need my blood pressure taken* […] *I need antibiotics or* […] *something along those lines.* *’* (Shaun, MSM, 41 years)

Despite presenting with HIV indicator conditions, five participants reported GPs missing their diagnosis, which undermined their confidence in them. For at least two of these, non-disclosure of relevant history to their GP may have contributed to missed opportunities for earlier diagnosis. Shaun (quoted above) disclosed to his GP about his drug use, but not his sexuality or his recent ‘sex binge‘. Although he had presented with a number of HIV indicator conditions, his GP did not offer HIV testing. Another participant, Will (also MSM), recalled feeling unable to disclose being unfaithful to his partner (who had the same GP), even when the GP asked him about sex in response to his symptoms. In contrast, four participants recalled it had been their GPs or practice nurses who had referred them for their initial HIV test.

Ultimately, the implied and sometimes explicit definition of a ‘good’ GP appeared to be seeing the same person each time, and that GP knowing and accepting the patient without judgment.

#### HIV knowledge

Some participants felt unsettled when GPs referred them back to clinics without preliminary investigation of their symptoms, which they felt demonstrated GPs’ lack of knowledge or confidence in dealing with HIV-positive patients:

*‘* *I think it’s deemed a manageable thing now, and so they think my GP is just as qualified, but I’m not always sure he is. I think he isn’t even that sure he is because he often just says,* *“* *I think you should ask your consultant.”…* *That leaves me feeling a bit in limbo.* *’* (George, MSM, 45 years)

However, others understood GPs could never have the specialist, up-to-date knowledge of HIV clinicians:

*‘ … with GPs. They can’t be experts on everything. They couldn’t possibly be.’* (Paul, heterosexual male, 56 years).

#### Agency

A few participants who felt ’in limbo‘ between the clinic and the GP took it upon themselves to coordinate care and communication, including obtaining and sharing their test results to offer that continuity. Others achieved continuity by ensuring they saw the same GP each time, even negotiating a double slot to allow sufficient time for the consultation. This was easier for non-urgent issues, but not always possible when they were unwell. Some of those who learned to make the system work for them became peer mentors for other PLHIV. Further examples of taking control included ‘shopping around‘ until they found suitable GPs or practices. For example, Eileen changed GPs because she felt judged:

*‘* *I remember when I joined* *... The very first GP I was with was when I was still living in* [location]*. She made such huge judgments about* [ex-boyfriend] *for infecting me I just thought,* *“* *I’m not going to be able to work with you.* *”* *So I then moved ... and got a new GP.* *’* (Eileen, heterosexual female, 45 years)

## Discussion

### Summary

The Positive Voices survey showed a high prevalence of GP registration and disclosure, both of which are fundamental to implementing a ’shared care‘ approach. Notably, this was across all groups, regardless of age, gender, sexual orientation, socioeconomic status (SES), or ethnicity. This suggests that the push from the HIV clinics to encourage registration with, and disclosure of status to, GP services has been effective. Similar to the general population,^15^ those with comorbidities and poor self-rated health status were more likely to report GP use, as were older adults and those of lower SES and financial insecurity. Nearly two-thirds of the sample reported satisfaction in the four GP PREMs. Nearly all participants in the qualitative study were also registered with a GP who knew about their HIV status. In the survey, the presence of non-HIV comorbidities was associated with higher recent GP attendance but not significantly better experiences, whereas in the qualitative study participants with long-term, non-acute health problems felt their GPs managed them well and were comfortable in their care.

In the survey, participants who reported avoiding health care due to their HIV status and those who worried about discrimination in healthcare settings were less likely to report positive GP experiences. While the healthcare context was not specified, and discrimination may not have occurred in primary care, any discrimination may influence attitudes towards GPs. In the qualitative study, participants discussed both actual and *anticipated* fears about using their GPs, suggesting that the key challenges for shared care are primarily about GP and practice staff enabling a trusting relationship to be built through offering sensitive and non-judgmental care, resembling that experienced in participants’ HIV services. Participants voiced concerns about GPs’ HIV knowledge, which were sometimes made worse by poor continuity of care and communication between primary and secondary care.

### Strengths and limitations

Combining study methods facilitates a more comprehensive understanding of a complex issue compared with using only one method. This study measured GP registration, disclosure, and satisfaction among PLHIV for the first time using a national survey, and interrogating these findings further using qualitative interviews helped to identify some practical steps to facilitate better care for PLHIV in the future.

The Positive Voices survey uses a large sample of PLHIV from across England and Wales, weighted to reflect the national distribution. However, the size of the dataset means that small differences were statistically significant, thus the absolute differences, effect sizes, and direction of the associations are more important when determining clinical significance. The qualitative sample was large and diverse in terms of ‘HIV generation‘ and demographics; it may not, however, be representative of experiences of PLHIV living in other locations. PLHIV living further away from large metropolises may have to travel greater distances to access HIV specialist clinics, and the authors suggest that encouraging the use of their local GP for their primary care needs may have some advantages in terms of reducing travel burden.

Finally, self-reports were not verified against documented clinical events and may be subject to responder bias. The authors who have worked as researchers and/or clinicians with PLHIV may have introduced some bias in the data collection and interpretation. Conversely, they might offer greater insights to enrich interview data and contextualise the findings.

### Comparison with existing literature

GPs are usually the first point of contact for people for primary health care advice and LTCs.^16^ For PLHIV, this role was and is often played by HIV specialists, and some are reluctant to make that shift.^6,17^ Without other LTCs, PLHIV may have little or no contact with a GP, sometimes causing apprehension. The present findings are consistent with a systematic review of aspects of health care most valued by PLHIV in high-income countries,^18^ which could be summarised to three main areas: a good, supportive practitioner–patient relationship involving patients in care decisions; access to specialist high quality HIV knowledge and support; and well-functioning care structures that provide effective, accessible continuity of care and services for all.

The fragmentation of HIV care following the Health and Social Care Act of 2012 resulted in uncertainties about how PLHIV should be cared for.^19,20^ As PLHIV age, the nature and scale of healthcare needs will become apparent; GPs already have skills and expertise in caring for people with LTCs, which could be transferable to PLHIV, but they need to build trusting relationships with them. Similar to another study, the present study found interview participants with non-acute LTCs developed a comfortable relationship with their GP, perhaps aided by more frequent contacts.^6^ A study from over 20 years ago reported that, while PLHIV were concerned about confidentiality, finding a ‘sympathetic and liberal attitude‘ was more important in deciding whether to consult their GP.^21^ PLHIV as a patient group are at particular risk of going through periods of acute vulnerability despite being ‘virally undetectable‘.^12^ Given the streamlining of HIV specialist services, GPs providing responsive and flexible care could be the essential service that supports PLHIV back to safety;^11^ however, pressure on GP time presents as a significant further challenge.

PLHIV’s apprehensions about GPs’ lack of specialist knowledge and their unfamiliarity with social and emotional aspects of life with HIV have been reported elsewhere.^5,6,22^ Some GPs and primary care teams could develop this knowledge and appropriate skills, but must be supported by training and recognition of time and resources.^5,9^ Concerns about GP–specialist communication and coordination of care are not new, and focus on two issues: first, GP concerns regarding complexity of ART medication, interactions, and side-effects^9^ mean that patients sometimes experience being ‘ping-ponged‘ between services;^20,22^ second, PLHIV’s own need for confidentiality about their HIV status makes them unsure about sharing data across wider care services,^8,17^ especially via primary care where patient data could be accessed by administration staff.^8^

### Implications for practice

Some of the issues faced by PLHIV are similar for patients with other LTCs; an NHS England report on improving health outcomes for people with LTCs identified lack of care coordination, lack of emotional and psychological support, fragmentation of care, lack of informational continuity, and poor care planning consultation as key problems.^23^ The solutions to these problems are also likely to be similar. In light of the advances in HIV treatment and high prevalence of GP registration and disclosure, GPs can be involved in managing the primary care needs of PLHIV, and can bring to the shared care model their expertise in prevention and screening for other chronic conditions, including mental health.

High levels of registration and a disclosure of status alone does not, however, necessarily translate into PLHIV using primary care services. ‘HIV-friendly‘ practices, or GPs who are seen to have knowledge of HIV and are sensitive to its social and psychological manifestations, can act as champions for greater PLHIV engagement. Individually, GPs might see few PLHIV and hence have limited experience, but simple steps could include raising HIV awareness and knowledge among GPs through training and updates on HIV prevention, treatment, and care (such as treatment-as-prevention [TasP] and its impact on sexual relationships and contraception). In areas of higher HIV prevalence, there may be justification for identifying a GP who becomes the ‘champion‘ (for a set of linked local primary care practices or clinical commissioning groups) for proactively managing the register and sharing knowledge with their colleagues.^24,25^

GPs might find it reassuring that PLHIV value skills such as empathy and person-centeredness — the same skills GPs use to manage other patients with LTCs and health problems in general practice. GPs showing willingness to share knowledge and consult specialists, and PLHIV could also promote trust and a mutually supportive partnership.

Perceived threats to confidentiality might be due to patients’ lack of awareness of data security and use of personal information, for which there are national standards for good practice and penalties for breaches.^26^ Practices should state their confidentiality obligations towards all patients and demonstrate their compliance by asking permission from the patient each time they wish to share their information with third parties as standard practice. PLHIV may also want reassurance that they can discuss their sexual health, and drug and alcohol use with GPs without fear of being judged.

The issue of stigma faced by PLHIV in healthcare settings requires a more proactive response to counter wider societal attitudes towards HIV. Practices can break down stigma by promoting and normalising HIV testing and the message ‘U equals U‘ or ‘undetectable equals untransmissible‘, which is the principle behind TasP.^27^ Publicly displayed messaging could help to reassure PLHIV that the practice is HIV-friendly and unlikely to discriminate against them.
